# Effect of Cadmium Chloride on Metallothionein Levels in Carp

**DOI:** 10.3390/s90604789

**Published:** 2009-06-17

**Authors:** Jana Kovarova, Rene Kizek, Vojtech Adam, Danka Harustiakova, Olga Celechovska, Zdenka Svobodova

**Affiliations:** 1 Department of Veterinary Public Health and Toxicology, Faculty of Veterinary Hygiene and Ecology, University of Veterinary and Pharmaceutical Sciences Brno, 612 43 Brno, Czech Republic; E-Mails: celechovskao@vfu.cz (O.C.); svobodovaz@vfu.cz (Z.S.); 2 Department of Chemistry and Biochemistry, Faculty of Agriculture, Mendel University of Agriculture and Forestry Brno, Zemedelska 1, 613 00 Brno, Czech Republic; E-Mails: kizek@sci.muni.cz (R.K.); vojtech.adam@mendelu.cz (V.A.); 3 Research Centre for Environmental Chemistry and Ecotoxicology, Faculty of Science, Masaryk University Brno, Kamenice 126/3, 625 00 Brno, Czech Republic; E-Mail: harustiakova@iba.muni.cz

**Keywords:** heavy metals, bioaccumulation, *Cyprinus carpio*

## Abstract

Due to anthropogenic activities, heavy metals still represent a threat for various trophic levels. If aquatic animals are exposed to heavy metals, we can obviously observe considerable toxicity. It is well known that organisms treated with heavy metals synthesize low molecular mass compounds rich in cysteine. In this work the effects of cadmium chloride (2.5, 5, 7.5, 10 and 12.5 mg/L) on common carp (*Cyprinus carpio*) was investigated. We determined cadmium content in tissue of muscle, liver and kidney by atomic absorption spectrometry with electrothermal atomization and content of metallothionein (MT) in the same tissues by the Brdicka reaction. Electrochemical methods can be considered as suitable and sensitive tools for MT determination in carp tissues. Results of our study showed a gradually enhancing of cadmium content in muscle with time and dose of cadmium chloride in water. MT levels in liver reached both high levels (above 130 ng/g) in fish exposed to 2.5, 5 and 7.5 mg/L and low level (to 50 ng/g) in fish exposed to 10 and 12.5 mg/L of cadmium chloride. This finding confirms that the synthesis of metallothioneins and binding capacity of these proteins is restricted.

## 1. Introduction

Cadmium (Cd) belongs to the group of highly toxic heavy metals. Naturally, it occurs in water only in trace amounts, but recently its levels have increased due to anthropogenic activities [1]. Most cadmium contamination comes from metal foundries, the dye industry, production of plastics and of accumulators. This exposure results in pathological changes in water ecosystems, mostly demonstrated in fishes, which are affected by heavy metals through the respiratory and digestive systems and through the skin. In general, toxic effects of all heavy metals are similar, including pathological changes in parenchymatous organs and the nervous system. Indeed, long-term exposure of cadmium has some specific effects like impairment of reproductive function and endocrine disruption. Current accepted opinion of cadmium action as well as other metals is related mainly to their influence on protein molecules, particularly enzymes. They have a strong affinity to bond with the aminoacid moieties of proteins and may cause changes in enzyme structures. The most obvious consequences of these changes are the inhibition of enzymes [2].

Detoxification systems for metals in plants, intervertebrates, fishes, birds and mammals are derived from the specific feature of stress induced molecules to bind heavy metals. Particularly it was discovered that organisms protect themselves against the toxic effect of metals by synthesis of extra proteins called metallothioneins (MTs), which are abundant throughout the whole animal kingdom [3]. These proteins are rich in cysteine and are able to bind metal ions [4]. MTs are a group of low molecular mass (6,000–7,000 Da) single-chain proteins, containing about 25–35% cysteine, due to which they have high binding capacity for metals. All SH-groups may bind a metal ion, however, about 50% of metal-binding sites are always saturated with Zn. One MT molecule can sequester 6-7 cadmium molecules [5]. In fish, MTs occur mainly in the liver, kidneys, gills and digestive tract [6]. Among fish species negligible differences in amino-acid composition, spectral characteristics and stoichiometric properties of MTs were found. Two MT isoforms corresponding to classes MT-I and MT-II (6,227 Da and 6,435 Da) were isolated from the hepatopancreas of the carp (*Cyprinus carpio*). These isoforms occur in trace amounts in cells, because their primary function is to maintain homeostasis of copper and zinc and the protection of cells against oxidative damage [7]. Nevertheless, sublethal concentrations of metals such as Cu, Cd, Ag or Hg induce their synthesis. Based on the data published by other authors [5,8,9], factors such as environmental stress, starvation, or bacterial infection may cause an increase in MT levels in animals, including fish. The molecular level of MTs is induced by interleukin-1, glucocorticoids, interferon and heavy metals as a matter of course [10].

For detection of metallothioneins, various analytical methods including spectrometry, liquid chromatography, capillary electrophoresis, and electrochemistry can be employed. In this study, a very sensitive technique, the differential pulse voltammetry Brdicka reaction was utilized for detection of this protein [11,12]. In our biological experiments, the influence of cadmium chloride in water on levels of cadmium in muscles, liver and kidneys and to levels of metallothioneins in these tissues of fish was studied.

## 2. Results and Discussion

### 2.1. Comparison of fish groups exposed to different concentration of cadmium chloride

Cadmium content in muscle, liver and kidney and MT in liver and kidney in fish groups exposed to different concentration of cadmium chloride for a specific period were compared by mean of Kruskal-Wallis test, followed by multiple comparison.

#### Muscles

**Figure 1.**
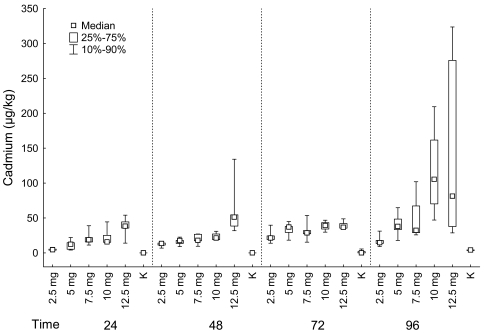
*MUSCLE*: Cadmium (μg/kg) in fish grouped according the exposition to cadmium chloride (mg/L) (K–control group).

Cadmium content in muscle differed significantly among fish groups exposed to different concentrations of cadmium chloride for 24, 48 and 72 hours, as well as for 96 hours (Kruskal-Wallis test: comparison of fish groups exposed for 24 hours: P < 0.001; comparison of fish groups exposed for 48 hours: P < 0.01; comparison of fish groups exposed for 72 hours: P = 0.05; comparison of fish groups exposed for 96 hours: P = 0.01). At all sampling times, cadmium content was the lowest in control group and the group exposed to 2.5 mg/L of cadmium chloride and increased in groups exposed to higher concentration of cadmium chloride (Figure 1).

Statistically significant differences in cadmium content in fish groups exposed to cadmium chloride for 24 hours were found between control group and groups exposed to 7.5, 10 and 12.5 mg/L of cadmium chloride (multiple comparison). Significant differences in cadmium content in fish groups exposed to cadmium chloride for 48 hours were found between the groups exposed to 2.5 mg/L and 12.5 mg/L of cadmium chloride, then between the control group and groups exposed to 10 mg/L and 12.5 mg/L of cadmium chloride (multiple comparison).

In groups exposed to cadmium chloride for 72 and 96 hours we found significant differences in cadmium content between the control group and groups exposed to 10 and 12.5 mg/L of cadmium chloride (multiple comparison).

#### Liver

**Figure 2.**
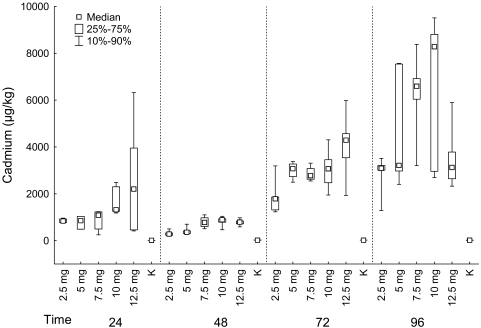
*LIVER:* Cadmium (μg/kg) in fish grouped according the exposition to cadmium chloride (mg/L) (K–control group).

Cadmium content and MTs in liver differed significantly among fish groups exposed to different concentrations of cadmium chloride for 24 hours, 48 hours, 72 hours as well as for 96 hours (Kruskal-Wallis test: *Cadmium*: 24 hours: P = 0.004; 48 hours: P = 0.001; 72 hours: P = 0.003; 96 hours: P = 0.006; *MTs*: 24 hours: P = 0.002; 48 hours: P = 0.005; 72 hours: P = 0.001; 96 hours: P = 0.003) (Figure 2).

For all sampling times, cadmium content was the lowest in the control group, then in the group exposed to 2.5 mg of cadmium chloride and increased in groups exposed to higher concentration of cadmium chloride. Significant differences among groups were found between control group and groups exposed to 10 and 12.5 mg/L of cadmium chloride (in groups exposed for 24 hours), between control group and groups exposed to 7.5, 10 and 12.5 mg/L of cadmium chloride (in groups exposed for 48 hours), between control group and group exposed to 12.5 mg/L of cadmium chloride (in groups exposed for 72 hours), between control group and groups exposed to 7.5 and 10 mg/L of cadmium chloride (in groups exposed for 96 hours) (multiple comparison).

**Figure 3.**
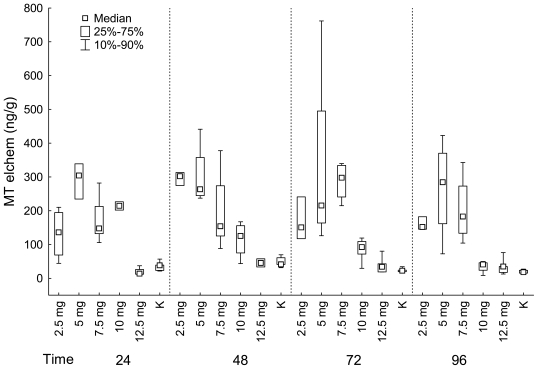
**LIVER:** MTs (ng/g) in fish grouped according the exposition to cadmium chloride (mg/L) (K–control group).

MTs in liver were generally the highest in groups exposed to 5 mg/L, 2.5 mg/L (in groups exposed to cadmium chloride for 48 hours), 7.5 mg/L (in groups exposed to cadmium chloride for 72 hours). The lowest values were found in control groups and groups exposed to 12.5 mg/L of cadmium chloride (Figure 3).

Significant differences among groups were found between control group and group exposed to 5 mg of cadmium chloride, between groups exposed to 5 mg/L and 12.5 mg/L of cadmium chloride (in groups exposed for 24 hours); between control group and groups exposed to 2.5 and 5 mg/L of cadmium chloride (in groups exposed for 48 hours); between control group and groups exposed to 5 mg/L and 7.5 mg/L of cadmium chloride, between groups exposed to 7.5 and 12.5 mg/L of cadmium chloride (in groups exposed for 72 hours); between control group and group exposed to 5 mg of cadmium chloride (in groups exposed for 96 hours) (multiple comparison).

#### Kidneys

**Figure 4.**
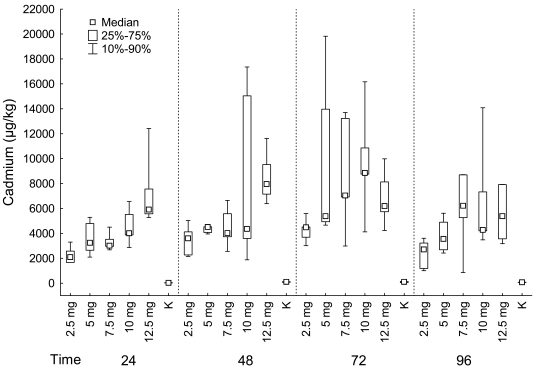
*KIDNEY*: Cadmium (μg/kg) in fish grouped according the exposition to cadmium chloride (mg/L) (K–control group).

Cadmium content in kidney differed significantly among fish groups exposed to different concentration of cadmium chloride for 24 hours, 48 hours, 72 hours as well as for 96 hours (Kruskal-Wallis test: *Cadmium*: 24 hours: P < 0.001; 48 hours: P = 0.003; 72 hours: P = 0.005; 96 hours: P = 0.004) (Figure 4).

**Figure 5.**
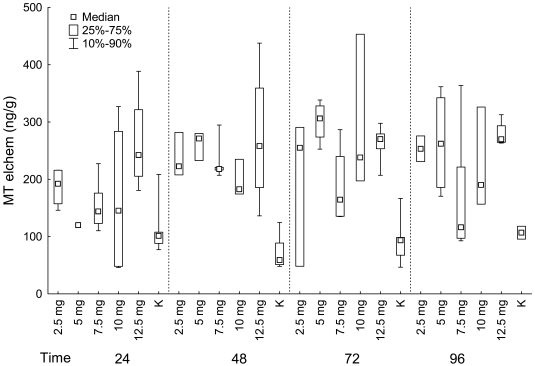
*KIDNEY:* MTs (ng/g) in fish grouped according the exposition to cadmium chloride (mg/L) (K–control group).

*MTs* in kidney differed significantly among groups exposed to different concentration of cadmium chloride for 48 and 72 hours (Kruskal-Wallis test: **MTs**: 24 hours: P = 0.100, in this comparison the group exposed to 5 mg/L of cadmium chloride was not included because of just one measure in this group; 48 hours: P = 0.020; 72 hours: P = 0.024; 96 hours: P = 0.280) (Figure 5).

For all sampling times, cadmium content was the lowest in control group, then in the group exposed to 2.5 mg/L of cadmium chloride and generally increased in groups exposed to higher concentration of cadmium chloride. Significant differences among groups were found between control group and groups exposed to 10 and 12.5 mg/L of cadmium chloride, between groups exposed to 2.5 and 10 mg/L (in groups exposed for 24 hours), between control group and group exposed to 12.5 mg/L of cadmium chloride (in groups exposed for 48 hours), between control group and groups exposed to 5 mg, 7.5 and 10 mg/L of cadmium chloride (in groups exposed for 72 hours), between control group and groups exposed to 7.5, 10 and 12 mg/L of cadmium chloride (in groups exposed for 96 hours) (multiple comparison).

**MTs** in kidney differed significantly among groups in fish exposed to cadmium chloride for 48 and 72 hours. MTs were found to be lowest in control groups. There was found a significant difference between control group and group exposed to 12.5 mg/L of cadmium chloride in fish exposed to it for 48 hours and a significant difference between control group and group exposed to 5 mg/L of cadmium chloride in fish exposed to it for 72 hours (multiple comparison).

### 2.2. Comparison of fish groups exposed to cadmium chloride for different time periods

#### Muscles

We found significant differences in cadmium content in muscle between groups exposed to cadmium chloride for different time period in groups exposed to 2.5, 5 and 10 mg/L of cadmium chloride and also in control group (Kruskal-Wallis test: 2.5 mg/L: P = 0.004; 5 mg/L: P = 0.007; 7.5 mg/L: P = 0.102; 10 mg/L: P = 0.005; 12.5 mg/L: P = 0.413; control group: P = 0.011).

In the fish exposed to 2.5 mg/L of cadmium chloride, we found the significant difference between groups exposed for 24 and 72 hours; cadmium content was higher in the group exposed for 72 hours (multiple comparison; Figure 1).

In the fish exposed to 5 mg/L of cadmium chloride, significant difference was found between groups exposed for 24 and 96 hours; cadmium content was higher in group exposed for 96 hours. In the fish exposed to 10 mg/L of cadmium chloride we found the significant difference between group exposed for 96 hours and groups exposed for 24 and 48 hours. The cadmium content was significantly higher in groups exposed for 96 hours than in groups exposed for 24 and 48 hours. In the control group, there was significantly higher cadmium content in muscle in group exposed for 96 hours than in group exposed for 24 hours (multiple comparison; Figure 1).

#### Liver

We found significant differences in cadmium content in liver between groups exposed to cadmium chloride for different time period in groups exposed to 2.5, 5, 7.5 and 10 mg/L of cadmium chloride and also in the control group (Kruskal-Wallis test: 2.5 mg/L: P = 0.002; 5 mg/L: P = 0.002; 7.5 mg/L: P = 0.003; 10 mg/L: P = 0.001; 12.5 mg/L: P = 0.051; control group: P = 0.009).

In the fish exposed to 2.5 mg of cadmium chloride, we found the significant difference between the group exposed for 48 hours and groups exposed for 72 and 96 hours; cadmium content was the lowest in the group exposed for 48 hours. In the fish exposed to 5 mg/L of cadmium chloride, the situation was the same as in the previous case. In fish exposed to 7.5 mg/L, significant differences were found between group exposed for 96 hours and groups exposed for 24 and 48 hours; being highest in the group exposed for 96 hours. In fish exposed to 10 mg/L of cadmium chloride, we found the significant difference between group exposed for 48 hours and groups exposed for 72 and 96 hours. The lowest value was found in the group exposed for 48 hours. In the control group, there was significantly lower cadmium content in liver in the group exposed for 24 hours than in groups exposed for 48 and 72 hours (multiple comparison; Figure 2).

We found significant differences in MTs in liver between groups exposed to cadmium chloride for different time period in group exposed to 10 mg/L of cadmium chloride and also in the control group (Kruskal-Wallis test: 2.5 mg: P = 0.083; 5 mg: P = 0.738; 7.5 mg: P = 0.383; 10 mg: P = 0.030; 12.5 mg: P = 0.297; control group: P = 0.012).

In the fish exposed to 10 mg/L of cadmium chloride, we found the significant difference between groups exposed for 24 and 96 hours; MTs in group exposed for 24 hours was higher. In the control group, there was a significant difference in MTs in liver between groups exposed for 48 and 96 hours; MTs in group exposed for 48 hours was higher (multiple comparison; Figure 3).

#### Kidneys

Cadmium content in kidney differed significantly between groups exposed to cadmium chloride for different time period in group exposed to 2.5 mg/L of cadmium chloride (Kruskal-Wallis test: 2.5 mg/L: P = 0.037; 5 mg/L: P = 0.062; 7.5 mg/L: P = 0.093; 10 mg/L: P = 0.315; 12.5 mg/L: P = 0.266; control group: P = 0.083), however the differences were not confirmed by mean of multiple comparison between any pair of groups (Figure 4).

There was no significant difference in MTs in kidney between groups exposed to cadmium chloride for different time period in any group exposed to cadmium chloride nor in control group (Kruskal-Wallis test: 2.5 mg/L: P = 0.361; 5 mg/L: P = 0.584; 7.5 mg/L: P = 0.381; 10 mg/L: P = 0.587; 12.5 mg/L: P = 0.811; control group: P = 0.410; Figure 5).

### 2.3. Correlation between cadmium content and MTs

Correlation between cadmium content and MTs in liver was not statistically significant in any but one group (fish group exposed to 12.5 mg/L of cadmium chloride for 24 hours; N = 4, r_s_ = -1.00, P < 0.001). The correlation was negative; increasing the cadmium content in liver, the MTs decreased. Correlation between cadmium content and MTs in kidney was not found to be statistically significant in most of the groups. The correlation between cadmium content and MTs was statistically significant and negative just in two groups: fish group exposed to 12.5 mg/L of cadmium chloride for 48 hours (N = 4, r_s_ = -1.00, P < 0.001) and fish group exposed to 5 mg/L of cadmium chloride for 72 hours (N = 4, r_s_ =1.00, P < 0.001).

Results of our study showed a gradual enhancement of cadmium content in muscle with time and concentration of cadmium chloride in water. Muscle is a type of tissue which is extrinsic to the target organs for cadmium accumulation and for metallothionein synthesis [13]. In our study, MTs level was nondetectable, thus level of cadmium in muscle is positive correlated with concentration of this ion in water.

In livers and kidneys the situation was different because of accumulation of this metal and because of enhancing of metallothionein synthesis in these tissues. The highest content of cadmium in kidneys was found already after 48 and 72 hours of exposure and in livers after 96 hours of exposure. However, it was reported that the first target organ for cadmium after exposure of cadmium salt in water is the liver, but the final target organs are kidneys [13-16]. Under lower concentrations of cadmium, we found higher concentration of cadmium in kidneys than in livers [17]. Higher concentrations of cadmium resulted in similar accumulation in liver and kidney tissues during the first several hours or days of the exposition. After longer time of exposure, the ratio is changed, where higher Cd levels were found in kidneys [17]. As mentioned above, cadmium may bind to MTs, displacing zinc. Cadmium presence inside a cell also may induce synthesis of new metallothioneins, and bind to it. Cadmium-MT complexes formed such way may be transported to the kidneys. Metal binding capacity depends on initial MTs level, and on intensity of further MTs synthesis. The most intense metallothioneins synthesis was usually observed in liver. Hepatic MTs concentrations in fish showed marked elevations in proportion to the metal concentrations lower than 10 mg/L. Intensity of MTs synthesis is, thus, tissue-specific, concentration and time-dependent. The ability of fish species to synthesize metallothioneins is also different among them. A difference between red-blooded and white-blooded Antarctic teleost was demonstrated. There the highest MTs contents were found in liver (short-time study of 60 minutes of exposure to high Cd concentration), and hepatic concentration of Cd, Cu and Zn and metallothioneins showed positive correlation in red-blooded teleosts (*Trematomus bernacchii*) but not in white-blooded teleosts (*Chionodraco hamatus*) [18]. Sex-differences in the synthesis of metallothioneins were demonstrated too. The levels of hepatic MTs in the male dab, *Limanda limanda*, were better correlated with cadmium exposure in the mixture with copper than in females [19]. The age of fish is other factor needs to be correlated. It is not surprising that the hepatic and kidney cadmium levels increase with the age of fish, due to accumulation of various metals. Similar behaviour was shown in metallothioneins concentration. This is consistent with findings that both Cd and MT concentration in fish tissue increase under conditions of long-term exposure to this metal [20-22]. In our study, MTs in liver reached high levels (above 130 ng/g) in fish exposed to 2.5, 5, and 7.5 mg/L of cadmium chloride and low levels (not more than 50 ng/g) in fish exposed to 12.5 mg/L cadmium chloride and in the control group. Similar trend could be seen in MTs in kidneys, although the decrease of MTs content in fish groups exposed to higher Cd concentration was not statistically significant. This finding could confirm the meaning that synthesis of metallothioneins and binding capacity of these proteins is restricted [23]. Furthermore, Dallinger *et al*. [23] observed that MTs isolated from livers and kidneys contained cadmium mainly and thus displaced zinc and copper. Moreover, they observed that metallothioneins induction usually needs some time to develop and cease after certain time of exposure [24]. It is obvious that MTs can detoxify certain concentration of cadmium only. Cadmium ions, which cannot be bind by MTs, interact with high molecular mass proteins. This phenomenon may result in toxic effects. Evidently, the cadmium portion not bound in metallothioneins [25-27] results in cadmium toxicity. Very similar results were presented in other study [28], in which it was shown that most of cadmium (60%) was located in the heat stable cytosolic component, probably bound by metallothioneins, protecting the liver from cadmium toxicity. In this study [29], the authors paid their attention to cadmium distribution in yellow perch (*Perca flavescens*) liver. Perches were under several natural long-term exposure of water-born cadmium. They showed that fish hepatic cellular components, fractionated by differential centrifugation, sequestered cadmium in constant ratios. Cadmium was bound by both cadmium-sensitive and resistant cellular components. Bio-accumulated amounts were in correlation with the exposure intensity.

It was demonstrated that induction of MTs synthesis by Cd depends on the way of metal uptake by the fish. The pathway for metal uptake in fish appears to be through gills, intestine and skin, but the relative extent of these ways varies, depending partly on the chemical and physical characteristics of water and sediments [28]. In the environment, metals are presented as free ions or as complexes with suspended particles and sediments. Transition metal ions dissolved in the ambient water are adsorbed through the gills [29] and other permeable body surface. Metals bounds to solid particles are ingested detached from their carrier particles in the digestive system and absorbed through the gut epithelium [30]. Experimental way for cadmium exposure as pharmacological injection way showed that absorption of cadmium by liver was much more efficient (17-18%) than through the physiological ingestion way (0.32-0.44%) in the sentinel fish (*Lithognathus mormyrus*) [31].

We show that MTs play an important role in cadmium detoxification in fish. Level of uptaken cadmium was closely related to the increase of MTs level. Correlation between cadmium content and metallothioneins concentration in tissues of liver and kidney were negative. This fact was statistically significant only in the following cases: i) in livers after 24 hours and doses of 12.5 mg/L and ii) in kidneys after 48 hours and doses of 12.5 mg/L and after 72 hours and doses of 5 mg/L. We should keep in mind, that the number of fish in each group was five individuals. It can be expected, that the correlations would be statistically significant in greater groups (ten or more fish). Same values were reported in the study of fish, where Cd was administered to the water for 29 days in four concentrations (0, 0.8, 4 and 20 μM). It was reported Cd accumulation in the tissues in following order: kidney > liver > gills. Concentration of Cd and Zn binding metallothioneins ((Cd, Zn)-MTs) were in the following order: liver > kidney > gills [32]. However, in another study [33] the authors showed that accumulation capacity of every single organ depends on other metals in water. Common carps co-exposed to cadmium, mercury and lead had the amounts of cadmium in this order: kidney > gills > liver > muscle and metallothioneins were in order: gills > kidney > liver > muscle. Detailed explanation of reason of this thing needs other studies.

## 3. Experimental Section

The effects of cadmium chloride (2.5; 5; 7.5; 10 and 12.5 mg/L) on 1-year old common carp (*Cyprinus carpio* L.) of average weight 78 ± 14 g was investigated. Carp in good health conditions were kept at aquariums where fthe ollowing basic physical and chemical indices of diluting water were used: temperature of water was 21 ± 1°C; oxygen saturation was above to 60% (ranging from 76 to 93%) and pH ranged from 7.8 to 8.4. ANC_4.5_ (acid neutralisation capacity) 3.56–3.75 mM; COD_Mn_ (chemical oxygen demand) 1.34–1.91 mg/L; total ammonia below detection limit; NO_3_^-^ 24.35–31.40 mg/L; NO_2_^-^ below detection limit, Cl^-^ 18.9–19.1 mg/L; sum of Ca ± Mg 14 mg/L. Water/tested solution in aquariums were exchanged daily. Fish (n = 5) from all experimental groups and control groups were sampled daily. From all sampled fish their liver, kidney and muscles were obtained. We determined content of cadmium in muscles, liver and kidney by atomic absorption spectrometry (AAS) with electrothermal atomization, and content of metallothionein in the same tissues by the Brdicka reaction.

### Cadmium in fish

Samples were mineralized by means of nitrogen acid and hydrogen peroxide in laboratory autoclaves with microwave heating (ETHOS SEL, Milestone Italy). The detection limits (3σ) for cadmium (Cd) was 0.1 μg/kg. Samples of reference materials BCR No 278 (muscle tissue) and IAEA MA-B-3/TM (fish homogenate) were used to check the validity and accuracy of the method. Concentrations of cadmium in tissues were determined by atomic absorption spectrometry (AAS) with electrothermal atomization (ZEEnit 700, Analytika Jena, D).

### Metallothioneins in fish

#### Stationary electrochemical analyser–Adsorptive transfer stripping differential pulse voltammetry Brdicka reaction

The sampled fishes (app. 0.2 g) were frozen with liquid nitrogen and spread in a mortar, and then exactly 1,000 μL of 0.2 M phosphate buffer (pH 7.2) was added to the homogenized sample. The obtained homogenate was transferred into test-tube and vortexed for 15 min at 4 °C (Vortex Genie, USA). The supernatant was subsequently heat-treated. Briefly, the sample was kept at 99 °C in a thermomixer (Eppendorf 5430, USA) for 15 min. with occasional stirring, and then cooled to 4 °C. The denatured homogenates were centrifuged at 4 °C, 15,000 g for 30 min. (Eppendorf 5402, USA). Heat treatment effectively denatures and removes high molecular weight proteins out from samples.

Electrochemical measurements were performed with AUTOLAB Analyzer (EcoChemie, Netherlands) connected to VA-Stand 663 (Metrohm, Switzerland), using a standard cell with three electrodes. Principle of the adsorptive transfer stripping technique (AdTS) is based on the strong adsorbing of the target molecule on the electrode surface at an open electrode circuit. The electrode is washed in a rinsing buffer. The electrode is further transferred to the supporting electrolyte and measured. The Brdicka supporting electrolyte containing 1 mM Co(NH_3_)_6_Cl_3_ and 1 M ammonia buffer (NH_3_(aq) + NH_4_Cl, pH = 9.6) was used and changed after five measurements; surface-active agent was not added. The samples of the MT were reduced before each measurement by 1 mM tris(2-carboxyethyl)phosphine addition. AdTS DPV Brdicka reaction parameters were as follows: an initial potential of -0.35 V, an end potential -1.8 V, a modulation time 0.057 s, a time interval 0.2 s, a step potential of 1.05 mV, a modulation amplitude of 250 mV, Eads = 0 V. All experiments were carried out at 4 °C (Julabo F12, Germany).

### Statistics

Cadmium content in muscle, liver and kidney and MTs in liver and kidney in fish groups exposed to different concentration of cadmium chloride for a specific period were compared by means of Kruskal-Wallis test followed by multiple comparison [34]. The same test was used to compare the cadmium content in muscle, liver and kidney and MTs in liver and kidney in fish groups exposed to specific cadmium chloride concentration for different time period.

The relationship between cadmium content and MTs in liver as well as between cadmium content and MT in kidney was examined by means of Spearman rank correlation [34]. The correlation coefficients were calculated separately for each fish group. All the tests were evaluated at the significance level α = 0.05. Data analyses were performed using Statistica software [35].

## 4. Conclusions

We determined that cadmium may bind to metallothioneins and that the metal binding capacity of these proteins is restricted. Available data show that metals may induce MT synthesis, which increases metal binding capacity, and the detoxification abilities of fish organism. Binding capacity depends not only on MT levels in the cells but also on possibility of their synthesis, which depends on many factors such as experimental body (fish species, age, gender), conditions of exposure (other metals in the water, characteristic of water medium), time period (acute/chronic type of exposure) and source of cadmium (concentration as well as chemical structure of compounds containing cadmium) etc.

In this study, we have confirmed the results of other authors [3] who concluded that the concentration of cellular stress proteins (including metallothioneins) is a good indicator of water pollution. However, it was reported that MT level was a good bioindicator of heavy metals pollution in *Salmo trutta*, but not in *Anguilla anguilla* [36]. Therefore, it could be concluded that not every fish species is suitable for biomonitoring. We have demonstrated herein that the common carp (*Cyprinus carpio*) could be considered as a specimen of fish suitable for this purpose.

## Figures and Tables

**Table 1.** Cadmium content in muscle, liver and kidney, MTs in liver and kidney in fish groups exposed to different concentration of cadmium chloride for a specific time period.

Fish group		Muscle		Liver				Kidney			
	Concentr.	Cd (μg/kg)		Cd (μg/kg)		MTs (ng/g)		Cd (μg/kg)		MTs (ng/g)	

Time		N	Median	N	Median	N	Median	N	Median	N	Median
24	2.5 mg	5	4.59	4	831.50	4	135.86	5	2092.00	4	192.16
24	5 mg	5	12.31	5	850.00	3	303.83	5	3237.00	1	120.03
24	7.5 mg	5	18.77	5	1081.00	5	147.66	5	3011.00	5	143.85
24	10 mg	5	15.77	5	1316.00	2	214.29	5	4003.00	4	145.26
24	12.5 mg	5	38.28	5	2200.00	4	13.76	5	5912.00	4	242.19
24	K	5	0.05	5	11.22	5	38.06	5	26.23	5	101.19
48	2.5 mg	5	13.54	5	279.00	3	302.07	5	3604.00	3	222.53
48	5 mg	5	17.74	5	355.00	4	263.19	5	4508.00	3	271.05
48	7.5 mg	5	18.02	4	771.00	5	153.67	5	4016.00	5	217.83
48	10 mg	4	21.41	5	890.00	4	125.22	5	4352.00	3	182.55
48	12.5 mg	5	51.19	5	790.00	2	45.52	5	7956.00	4	257.84
48	K	5	0.05	5	18.57	5	40.99	5	105.60	5	58.82
72	2.5 mg	5	21.43	5	1782.00	3	150.40	5	4488.00	3	254.99
72	5 mg	5	37.01	4	3069.00	4	215.24	5	5376.00	4	306.36
72	7.5 mg	5	28.53	4	2773.00	4	297.48	5	7033.00	4	164.41
72	10 mg	4	39.26	5	3067.00	5	92.13	5	8841.00	3	238.00
72	12.5 mg	5	36.73	5	4288.00	5	33.92	5	6196.00	5	270.14
72	K	5	0.05	5	21.49	5	22.18	5	103.10	5	93.53
96	2.5 mg	5	14.64	5	3088.00	3	151.57	5	2705.00	2	253.27
96	5 mg	5	38.01	5	3209.00	4	284.21	4	3560.00	4	262.08
96	7.5 mg	4	32.47	5	6589.00	4	182.84	5	6213.00	5	116.30
96	10 mg	5	105.46	5	8276.00	4	40.57	5	4268.00	3	190.16
96	12.5 mg	5	81.18	5	3121.00	5	34.58	5	5370.00	4	270.01
96	K	5	4.02	5	15.83	5	17.99	5	70.75	2	106.83
